# How to Investigate Within-Subject Associations between Physical Activity and Momentary Affective States in Everyday Life: A Position Statement Based on a Literature Overview

**DOI:** 10.3389/fpsyg.2013.00187

**Published:** 2013-04-29

**Authors:** Martina K. Kanning, Ulrich W. Ebner-Priemer, Wolfgang Michael Schlicht

**Affiliations:** ^1^Division I Sport and Health Science, Department of Sport and Exercise Science, University of StuttgartStuttgart, Germany; ^2^Karlsruhe Institute of TechnologyKarlsruhe, Germany; ^3^Central Institute of Mental Health MannheimMannheim, Germany

**Keywords:** ambulatory assessment, physical activity, affective states, methodological requirements, ecological momentary assessment

## Abstract

Several meta-analyses have investigated the association between physical activity and affective states and have found evidence suggesting that exercise exerts a positive effect on affective state. However, in this field of research, most studies have conducted between-subject analyses. Nonetheless, there is more and more interest in the within-subject associations between physical activity and momentary affective states in everyday life. This position statement pertains to this up-and-coming field of research and provides methodological recommendations for further studies. The paper is divided into three parts: first, we summarize and evaluate three methodological requirements necessary for the proper evaluation of within-subject associations between physical activity and momentary affective states in everyday life. We propose that the following issues should be considered: (a) to address the dynamic nature of such relationships, repeated assessments are necessary; (b) as activities performed in everyday life are mostly spontaneous and unconscious, an objective assessment of physical activity is useful; (c) given that recall of affective states is often affected by systematic distortions, real-time assessment is preferable. In sum, we suggest the use of ambulatory assessment techniques, and more specifically the combination of accelerometer-assessment of physical activity with an electronic diary assessment of the momentary affective state and additional context information. Second, we summarize 22 empirical studies published between 1980 and 2012 using ambulatory assessment to investigate within-subject associations between momentary affective states and physical activity in everyday life. Generally, the literature overview detects a positive association, which appears stronger among those studies that were of high methodological quality. Third, we propose the use of ambulatory assessment intervention (AAIs) strategies to change people’s behavior and to enable people to be active as often as possible during the day (e.g., reducing sitting time, taking more steps per day).

## Introduction

Several meta-analyses (e.g., Arent et al., 2000; Netz et al., 2005; Puetz et al., 2006; Reed and Ones, 2006; Netz, 2009; Reed and Buck, 2009) have consistently revealed a positive between-subjects relation between physical activity and affective states; that is, a range of people feel better after having been physically active. To clarify whether physical activity might be a useful strategy to enhance good feelings, it is necessary to additionally investigate the within-subject relation; that is, does an individual person feel better after having been physically active? Unfortunately, the within-subject relations between these parameters have not been studied intensively. However, technological progress in recent years, especially in the field of mobile technology and statistics, enables the investigation of this relation with much higher precision than ever before. Accordingly, the purpose of this paper is to instruct readers regarding this new and innovative field of research on assessing the within-subject associations between physical activity and momentary affective states in everyday life according to the highest methodological standards.

In this position statement, we will propose, after providing an introduction to the assessment of physical activity and affect in general, three methodological standards, namely, (a) the need to address the dynamic process of the association; (b) the objective assessment of physical activity; and (c) the importance of real-time assessment. We suggest that meeting these standards is important in future studies. Second, we will report and summarize studies investigating the within-subject association between physical activity and momentary affective states in everyday life and will discuss whether they meet the methodological requirements of objective assessment and real-time assessment. Results will be summarized and discussed. Third, we will discuss how intervention studies can make use of the new methods.

### Physical activity

Physical activity is an umbrella term. There are different categories that fit under this term, ranging from playing sports or engaging in exercise at high levels of intensity to ambulating, which falls at the other end of the intensity scale. The latter category includes non-exercise activity thermogenesis (NEAT; Levine et al., 2005) or – in the vocabulary of gerontology and geriatrics – the basic and instrumental activities of daily living (ADL).

Physical activities can also differ according to the context in which they occur. Specifically, they can be performed in the workplace, during leisure time, by commuting, or by keeping the house clean. Furthermore, a person can be physically active for several reasons and for different motives (e.g., to meet other people, to strengthen health, or to run errands).

The physical activities of everyday life include in addition to sports and exercise mainly activities, which are done to achieve an intended purpose. For instance, people walk to the railway station, do gardening, walk to the supermarket, bike for transport, or play badminton with their children. In contrast to structured and regular exercise, the majority of everyday life activities is often processed automatically and habitually or performed spontaneously. The physical activities of everyday life include both categories of activities, and we label these activities here as “actual physical activities” (aPA).

Although aPA includes spontaneous and regular physical activities, there are certain important ways in which these kinds of activities differ. Compared with spontaneous activities, regular exercise often has a planned structure, will be repeated at a certain time and primarily includes higher levels of energy consumption. In most cases, regular exercise lasts longer, and people are often active together with others. However, spontaneous or habitually performed activities in everyday life may be planned (e.g., walking with the dog every day), but they may not be as stringent regarding time, place, and duration, in contrast to regular exercise or participation in sports. These activities during everyday life can last for a long (e.g., a bicycle tour during the weekend) or a short (e.g., walking to the railway station) period, and a person may be physically active on his or her own or together with others. Thus, spontaneous or habitually performed activities are more flexible than regular exercise. The person can be physically active in a way he or she likes the most at the moment. Therewith, these activities may have a subtle influence on a person’s affective state.

### Affective states

Definitions of emotion, mood, and affect are not universally accepted, and, as Smith and Lazarus (1990) proposed, these constructs make up an “inherently fuzzy set” (p. 611). Thus, it is imperative that researchers use theoretically grounded definitions of the affective constructs and adequate instruments to measure affective states of interest. Affective states could be measured as a general construct (on a trait level) or as a momentarily construct (on a state level). In this position statement, we refer to momentary affective states as an elementary, conscious accessible feeling that could be good or bad and of high or low arousal. Affective states are irreducible and similar to the term “core affect” of Russell (1980) and they are most general compared to mood and emotion.

Unfortunately, an intense discussion about differences in the theoretical constructs of mood, affect, and emotions is beyond the scope of this paper. For a deeper discussion, readers are referred to Cabanac, 2002; Frijda, 1994; Scherer, 2005, 2009; and Russell, 2003 or to Ekkekakis and Petruzello, 2000 for a detailed discussion about the differences between affective constructs in the domain of Sport and Exercise Psychology.

### Within-subject relations versus between-subject relations

**Figure 1:**
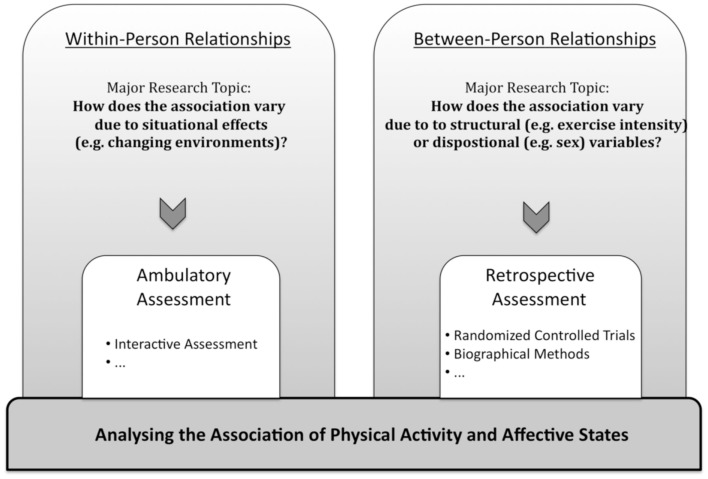
**Distinction between within-subject and between-subjects relationships in major research topics and assessment approaches**.

Comparable to the spontaneous or habitually performed activities of everyday life, momentary affective states on a state level are a volatile phenomenon. Momentary affective states show labile-state characteristics, whose dynamic quality is of interest (Ong et al., 2007). Therefore, it would be worthwhile to investigate intra-individual changes in addition to inter-individual differences or general effects following exercise or sport participation. Analyses of within-subject relationships reveal more the subtle and immediate effects of momentary affective states on physical activity, and vice versa, than is possible through analyses of between-subjects relationships. To fully understand the difference between within and between-subjects relationships, it is important to keep in mind that one cannot draw within-subject conclusions from across-person associations (Shiffman et al., 2008; Hamaker, 2012). Moreover, results based on within-subject data may contradict findings from between-subjects studies. For example, one result from some meta-analyses referring to intervention studies (e.g., Netz et al., 2005; Reed and Ones, 2006) is that regular exercise (on a trait level) has positive and significant effects on general affective states (on a trait level). The researchers tried to examine whether people who engage in low volumes of physical activity differ from those people who engage in high volumes of physical activity in their affective states. These findings do not imply that a person feels positive after having been physically active or feels negative (both on a state level) after having been inactive. Researchers who are interested in how much people vary over time in affective states and to what extent this variation is affected by physical activities should assess individuals during everyday life repeatedly and over time (see Figure 1). This process is necessary because inferences about the experiences of an individual cannot be made without observing that individual when he or she is actually physically active or not (Hektner et al., 2006).

In summary, analyses of between-subjects relations yield knowledge of important structural, dispositional variables that distinguish persons from one another, whereas analyses of within-subject relations yield insights into the dynamic association between variables and their dependence on situational circumstances (Bolger et al., 2003). To address the dynamic process between affective states and daily physical activity, different participants should be assessed repeatedly over time, during everyday life (Ebner-Priemer and Trull, 2009).

### Studies and meta-analyses on the association between physical activity and affective states

Most studies investigating the effects of physical activity on affective reactions have focused on structured activities, such as exercising or sports that were engaged in during leisure time to strengthen health or simply for recreational purposes. Several meta-analyses have shown that physical activity is positively associated with affective states and well-being, although convincing theoretical models of the association between physical activity and affective states are missing so far (e.g., Raglin et al., 2007). Looking only at the last decade, a narrative review (Netz, 2009) and two systematic meta-analyses (Arent et al., 2000; Netz et al., 2005) assessed the link between regular exercise and subjective well-being and mood in older adults without clinical disorders. According to the reviewed results, older people reported higher values of well-being post-exercise compared to baseline (for the intervention groups). In addition, improved mood and sense of well-being are associated with regular exercise. A further meta-analysis performed by Puetz et al. (2006) found similar associations between regular exercise and feelings of energy and fatigue. Regular exercise refers to cumulative bouts of structured exercises intended to improve physical fitness. Approximately 80% of the effect sizes reported in their meta-analysis were based on patients’ data (persons suffering from cancer or chronic fatigue, involved in cardiac rehabilitation, or suffering from psychic disorders, such as anxiety or depression). In addition, two meta-analyses investigated the effects of acute (Reed and Ones, 2006) and regular (Reed and Buck, 2009) exercise on positive affect. Both analyses showed that a person’s odds of feeling better increase after engaging in activities of low-to-moderate intensity for at least 30 min all at once.

The aforementioned meta-analyses examined the effect of structured exercise interventions or the effect of precisely described physical activity on different constructs of mental health (on a trait level). In addition, they analyzed moderator effects related to exercise conditions, such as intensity and duration or related to the baseline values of affective states. These studies did not analyze, if individual’s affective states vary due to physically active or inactive situations in every day life. Therefore, the findings did not show whether physical activity has a subtle influence on person’s affective states in everyday life.

In addition, many studies have assessed affective states during everyday life with a set of items referring to positive affective states, such as “cheerful,” “happy,” and “joyful,” or negative affective states, such as “sad,” “angry,” “stressed,” and “depressed.” The authors of these studies lacked a theoretical framework of affective states, and they did not specify what they intended to measure. With such a broad perspective, affective constructs such as mood, affect, feelings, and emotions were considered to be synonymous. However, the constructs designated by these terms differ along several dimensions, such as duration and origin, for instance. Furthermore, an important distinction between affective constructs is whether a relation between the subject and a particular object or event is involved (cf. Frijda, 1994).

## Methodological Requirements for Investigating Within-Subject Associations

### Assessments should be done repeatedly in real time

Repeated real-time assessments provide the opportunity to conduct context-sensitive analyses, whereas retrospective and cross-sectional reports, such as questionnaires or interviews, cannot precisely assess time-dependent processes and are limited in revealing context-sensitive information. Episodes of physical activity may be triggered by situational cues, whether external (e.g., a call from a friend, the local weather, or other environmental cues) or internal (e.g., cognitive cues or memories). Gaining a better understanding of episodes of physical activity, including their antecedents and consequences, is of theoretical and practical importance. *Do I feel better after running? Or do I exercise more when I’m feeling well and energized?* Such individual information supports understanding of the patterns that determine a person’s physical activity and may be used for interventions to increase physical activity.

Experimental data, autobiographical studies, and investigations of daily life have all demonstrated that retrospective assessments are a highly dubious methodology (Stone et al., 2002; Fahrenberg et al., 2007), given that people’s recall is vulnerable to multiple, systematic distortions and is often based on biased storage and recollection of memories (Fredrickson, 2000). Multiple memory heuristics have already been identified. For example, findings suggest that information associated with positive affect is more easily remembered than that associated with negative affect (i.e., the *affective valence effect*). However, the *mood-congruent memory effect*, the *peak end rule*, and *duration neglect* not only increase inaccuracy but also introduce systematic errors (Ebner-Priemer and Trull, 2009). The U.S. Food and Drug Administration fostered a discussion on heuristics, biases, and distortions by informing the pharmaceutical industry that real-time data are desirable because retrospective reports may be biased (U.S. Department of Health and Human Services Food and Drug Administration, 2009).

Real-life assessment is of special importance, given that multiple studies have shown that behavior (e.g., physical activity) that is manifested and measured under laboratory conditions is not representative of behavior performed in daily life outside of the laboratory. A relevant example of this distortion was reported by Horemans et al. (2005). The authors found that the heart rate of post-polio patients while walking at a self-paced speed was significantly lower in a laboratory compared to what it was in daily life; however, the same was not true for step rate. This difference is likely due to the more demanding environment of daily life (e.g., dual- or multi-tasking, uneven ground) and calls into question the generalizability of laboratory-based measurements. For a more detailed discussion of laboratory and real-world differences and the distinction between performance (“do do”) and capacity (“can do”), please see Bussmann and Ebner-Priemer (2011).

### Physical activity should be measured objectively

In recent years, exercise and health sciences have experienced a tremendous increase in the use of objective methods to assess physical activity (see, for example, a special issue in Medicine and Science in Sports and Exercise, Volume 44, Supplement 1, January 2012). Multiple types of accelerative devices, especially accelerometers, are used all over the world to capture the amount of physical activity subjects are performing in everyday life. However, there are studies and papers that employ a methodological shortcut, namely, retrospective questionnaires, when assessing the unconscious activities of daily life (spontaneous or habitually performed). Instead of actually assessing the amount of physical activity performed, such studies allow participants to estimate their typical level of physical activity or the number of minutes of vigorous activity in which they engaged during the last 2 weeks. From a memory perspective, we would not assume a high level of precision in such estimations. Accordingly, several authors question whether self-assessments of physical activity can serve as a substitute for the collection of actual behavioral data in everyday life (Baumeister et al., 2007; Fahrenberg et al., 2007) and caution against studies relying solely on self-reported physical activity (Ward et al., 2005; de Vries et al., 2006; Rapport et al., 2006).

Two systematic reviews give empirical evidence that objective assessment and subjective ratings of physical activity are not closely related. Prince et al. (2008) analyzed 187 studies that assessed physical activity both objectively (directly measured, primarily through accelerometry) and subjectively (self-reports, e.g., questionnaires or diaries). Overall, the correlations were low-to-moderate, with a mean of 0.37, even though the same construct (physical activity) was measured by the two different assessment methods. Similarly, Adamo et al. (2008) found low-to-moderate associations (−0.56 to 0.89) when comparing 83 studies using self-reports versus direct measures in a systematic review of physical activity in children. In both reviews (Adamo et al., 2008; Prince et al., 2008), self-report measures of physical activity had generally higher results than objective measures, and self-reports overestimated physical activity to a greater extent in females than in males. Taken together, the findings show substantial discrepancies and only moderate correlations between self-reports and direct measures, suggesting that the measurement method does have a significant impact on the data that are revealed. Consequently, Adamo et al. (2008) and Prince et al. (2008) question the widespread justification of using more cost-effective methods by positing that there are meaningful correlations between indirect and direct measures.

However, objective measures also have their limitations and cannot be viewed uncritically as the gold standard. Whereas most activities are captured quite easily (e.g., whole body movement), other forms of activities are quite difficult. In particular, situations in which participants sit and perform a physical activity (e.g., cycling, certain forms of weightlifting exercise) are typically underestimated. However, new software algorithms help to accurately classify movement patterns (e.g., cycling, taking the bus) and, in so doing, enhance the precision of estimating energy consumption.

Therefore, we recommend our second methodological requirement, that unconscious activities (spontaneous or habitually performed) should be measured objectively.

### Affective states should be measured electronically to ensure compliance

The third methodological requirement addresses the electronic assessment of affective states. Two methodological issues require further consideration. First, there is some evidence that the length of the recall interval determines the amount of report inaccuracy. Broderick et al. (2008) reported data showing that an increase in the recall period from 1 to 7 days was accompanied by a consistent increase in recalled reports of pain, although e-diary ratings did not increase over time. This finding implies that shorter recall intervals should facilitate the gathering of meaningful and reliable data. Second, an assessment can only be called real time if the procedures used can evaluate timely compliance to prompts, such as electronic diary devices that time-stamp responses. This aspect is important, as Stone et al. (2002) demonstrated in a paper-pencil diary study in which most participants reported themselves to be compliant 90% of the time. However, checking compliance by objective light sensors revealed that only a minimal number of reports were completed according to the time schedule set by the experimenters (11%). Therefore, we recommend electronic equipment for data acquisition to circumvent “back-filling,” in which assessment points are completed en masse immediately prior to a visit to the researcher (for a detailed discussion of the pros and cons of electronic versus paper-pencil diaries, see Stone et al., 2002; Green et al., 2006; Tennen et al., 2006).

To summarize, we referred in this section to three methodological requirements to analyze within-subject associations between physical activity and affective states. Studies of high methodological quality (a) assess the variables repeatedly in real time in everyday life, (b) measure physical activities of everyday life objectively, and (c) measure affective states with self-reports electronically to capture the data with maximum accuracy.

Ambulatory assessment is a promising method for addressing these abovementioned methodological requirements.

Different terms have been used for methods that capture data repeatedly in real life and real time, including ambulatory assessment (Fahrenberg et al., 2007), experience sampling (Csikszentmihalyi and Larson, 1987), and ecological momentary assessment (Stone et al., 2007). To simplify matters, we will use the term ambulatory assessment, which is defined by the Society for Ambulatory Assessment as follows: “*Ambulatory Assessment* comprises the use of field methods to assess the ongoing behavior, physiology, experience, and environmental aspects of humans or non-human primates in naturalistic or unconstrained settings. Ambulatory Assessment designates an ecologically relevant assessment perspective that aims at understanding biopsychosocial processes as they naturally unfold in time and in context.” (www.ambulatory-assessment.org).

## Studies on Within-Subject Associations between Physical Activity and Affective States in Everyday Life: An Literature Overview

### Literature search

To gain an overview of the proposed research field, we did a rough literature search strategy. We searched for studies analyzing within-subject associations between physical activity and momentary affective states assessed in real time in everyday life.

Our inclusion criteria were as follows:

1. To find studies assessing the aforementioned relation in everyday life, we included studies that used one of the following key terms: accelerometry, ambulatory assessment, ambulatory monitoring, computer-assisted diary, ecological momentary assessment, ecological momentary intervention, electronic diary, experience sampling method, hand-held computer.
2. To find studies that assessed physical activity, we included studies that used one of the following key terms: ADL, exercise, NEATs, physical activity, sedentariness, sport.
3. To find studies that assessed momentary affective states, we included studies that used one of the following key terms: affect, affective reactions, affective states, emotions, feelings, mood.
4. In addition, we included English-language studies that were published between 1980 and June 2012.

All four inclusion criteria had to be met for a study to be included. Reading the abstract, we checked if the relation between physical activity and affect was investigated as a within-subject relation (inclusion criteria five) and if the study was performed in everyday life (inclusion criteria six), i.e., not in a laboratory setting.

The exclusion criteria were as follows:

a) Studies that measured affect as a trait variable and not through multiple measurements in the individual’s natural environment (e.g., the better aging project; Fox et al., 2007; Parfitt et al., 2009) were excluded.
b) Studies that did not assess momentary affective states or aPA in real time, but with global diary surveys that were completed daily, for instance, shortly before bedtime (e.g., Giacobbi et al., 2005; Hyde et al., 2011; Poole et al., 2011) were excluded. In these studies, affective states and aPA are represented by retrospective evaluations.

We conducted computer searches in several relevant scientific databases (PubMed, Psyndex, PsycInfo, and Google Scholar). *All in all*, we found 393 articles. Abstracts were read, and all potentially relevant full manuscripts were retrieved (*N* = 24). Due to the exclusion criteria mentioned above, we excluded five studies. Next, the reference lists of the retrieved articles were searched for additional pertinent studies. This search yielded three additional studies, totaling 22 publications.

### Descriptive reports

In the 22 publications, within-subject associations between momentary affective states and aPA in daily life were investigated. Two publications referred to the same study (Schwerdtfeger et al., 2008, 2010). A total of 1799 individuals participated (1356 females, 418 males). The ages ranged from 10 to 85 years; thus, the studies assessed nearly all age groups, from adolescents (Axelson et al., 2003; Bohnert et al., 2009; Dunton et al., 2011) to young adults (Gauvin and Szabo, 1992; Vansteelandt et al., 2007; Hausenblas et al., 2008; LePage and Crowther, 2010; Kanning et al., 2012) to middle-aged and older people (the remaining 14 studies). In most cases, the sample was a healthy cohort; however, seven studies dealt with patient groups with conditions such as affective or eating disorders (Axelson et al., 2003; Vansteelandt et al., 2007), breast cancer (Grossman et al., 2008), joint replacement (Powell et al., 2009), knee osteoarthritis (Focht et al., 2004), or chronic pain (Vendrig and Lousberg, 1997). Two studies included overweight people (Carels et al., 2007; Rofey et al., 2010). Although all of the studies made assessments in real time, the number of time points of measurement per day ranged from 1 to 30. Likewise, the study periods ranged from 12 h to 70 days. All studies took place in the context of everyday life.

We used the second (physical activity should be measured objectively) and third (affective states should be measured electronically) methodological requirements to structure and evaluate the methodological quality of the 22 publications. First, in seven publications describing six studies, aPA was assessed objectively with accelerometers, and due to the use of electronic diaries or telephone calls, it was possible to determine the timing of the diary entries. In so doing, retrospective bias was minimized.

Second, in four studies, either aPA was assessed objectively, or the time points of the measurements were controllable. Third, in the remaining 11 studies, aPA was not assessed objectively, nor were the time points of measurements controllable by means such as electronic diaries.

### aPA was assessed objectively and affective states were assessed with electronic devices

The results of six articles (Grossman et al., 2008; Schwerdtfeger et al., 2008, 2010; Powell et al., 2009; Dunton et al., 2011; Kanning et al., 2012) showed that aPA and momentary affective states were significantly and positively associated. However, Axelson et al. (2003) arranged a feasibility study and did not report statistical analyses. Two studies (Grossman et al., 2008; Dunton et al., 2011) used a set of items to measure positive and negative affects, whereas the remaining studies assessed affective states with the Activation-Deactivation Adjective Checklist (AD-ACL; Thayer, 1989), a Mood Scale (Wilhelm and Schoebi, 2007), or the Positive and Negative Affect Schedule (PANAS; Watson and Telegen, 1988) (see Table 1).

**Table 1:** **aPA was assessed objectively and affective states were assessed electronically**.

Reference	Aim of the study	Sample	Procedures	Measurements		Results
				Physical activity	Affective states	
Axelson et al. (2003)	Pilot study: testing feasibility to perform ambulatory assessment with symptomatic patients with pediatric disorders	16 Children with affective disorders (major depressive disorder, generalized anxiety disorder, bipolar disorders) + 5 healthy controls, 9 girls, 12 boys, 10–17 years (*M* = 14.4; SD = 1.6)	Five extended weekends, Pbn received telephone calls – 12 calls between 4:00 p.m. (Friday) and 10:00 p.m. (Monday)	Self-report + accelerometer: ActiGraph, on wrist, 60 s epoch	Subset of PANAS-C, four positive (happy, joyful, exited, energetic) and four negative items (sad, angry, nervous, upset)	Performing ambulatory assessment for real-time experience sampling is feasible in symptomatic patients with pediatric affective disorders. Statistical analyses were not performed
Dunton et al. (2011)	To determine whether leisure time physical activity levels and experiences differ across social and physical contexts among children	121 Children (62 male), 9–13 years	4 days (Friday 4:00 p.m. to Monday 8:30 p.m. – not during school hours) random time within seven pre-established intervals, mobile phone, electronic diary	Accelerometer: ActiGraph (7164 GT2M), right hip, 30 s epoch	Electronic diary; positive affect: happy + joyful, negative affect: sad, angry, stressed, anxious	Affect differed during physical activity across physical and social contexts: greater ratings of positive affect when physically active outdoors, greater ratings of negative affect when physically active alone and with family only
Grossman et al. (2008)	To compare activity and mood between post-treatment breast cancer patients and matched control females	33 Post-treatment breast cancer patients + 33 healthy controls, age: *M* = 51.2; SD = 10.2	One weekday, every 50 min during awake hours	Accelerometer: LifeShirt	Electronic diary; mood (happy, sad, angry, anxious)	Activity did not differ between groups. Cancer patients were less happy across the day than healthy controls. Averaged accelerometry activity was correlated with mean self-reported energy and happiness
Kanning et al. (2012)	To analyze the effect of actual physical activity, autonomous regulation mode, and their interaction on affective states	44 University students (21 female), age: *M* = 26.2; SD = 3.2	One weekday, every 45 min between 8:00 a.m. and 10:00 p.m.	Accelerometer: Varioport-e, right hip, 60 s epoch	Electronic diary; short scale; six bipolar adjectives measuring valence, energetic arousal, and calmness	Actual physical activity, autonomous regulation mode, and their interaction significantly influenced affective states
Powell et al. (2009)	To explore the associations of negative and positive affect with activity levels using ecological momentary assessment	25 Individuals (36% female), 46–85 years old (*M* = 71.4) who had undergone total joint replacement of either knee or hip 12 months earlier	2-day study, diary sounded an alarm every 90–120 min from 9:00 a.m. until the participants went to bed	Self-report: computerized diary records, objective activity assessment: accelerometer: Vitaport 3, on trunk (lower part of sternum) and thighs	Electronic diary; positive affect; cheerful, negative affect; irritable, depressed, anxious, frustrated; PANAS on the following day	Walking time and dynamic activity was associated with lower negative affect. More activity was also associated with higher positive affect, however only the correlation of self-reported walking time with PANAS positive affect reached significance
Schwerdtfeger et al. (2008)	Is there a correlation between everyday life physical activity and psychological well-being?	124 Volunteers (64 females), 18–73 years old *M* = 31.67, SD = 12.56; BMI: *M* = 23.23; SD = 3.14	12 h Study on a typical workday, averaged bodily movement across four time windows (1, 1–5, 1–15, 1–30 min before assessment of affect)	Accelerometer: ActiGraph (GT1M), on left ankle	Electronic diary; adopted version (German version) of PANAS and AD-ACL to assess positive and negative affect	Daily physical activity episodes were associated with positive affective states not with negative affective states
Schwerdtfeger et al. (2010)	To examine whether momentarily assessed affect and bodily movement in everyday life are mutually associated	124 Volunteers (64 females), 18–73 years old *M* = 31.67, SD = 12.56; BMI: *M* = 23.23; SD = 3.14	12 h Study on a typical workday, averaged bodily movement across four time windows (1, 1–5, 1–15, 1–30 min before and after assessment of affect)	Accelerometer: ActiGraph (GT1M), on left ankle	Electronic diary; adopted version (German version) of PANAS and AD-ACL to assess positive and negative affect	Affective states and physical activity in every day life were mutually associated

### Either aPA was assessed objectively or affective states were assessed with electronic devices

Dunton et al. (2009) showed in their pilot study with 23 older adults that positive affects had significant positive impacts and negative affects had significant negative impacts on the total minutes of moderate-to-vigorous physical activity. However, participants self-reported their aPAs. Rofey et al. (2010) performed a feasibility study with 20 adolescents to analyze behaviors (e.g., aPA) and emotions during everyday life. To elicit ambulatory data, participants received telephone calls from trained staff members conducting a structured interview to evaluate current aPA and affective states. Thus, the time point of data entry was controllable, but aPA was assessed with self-reports. The authors did not report statistical analyses. McCormick et al. (2008) used accelerometry to assess aPA objectively, but they assessed psychological variables with a “paper-pencil” method using a pager to receive repeated self-reports during everyday life. The study of Vansteelandt et al. (2007) was the only one in this section that used a theoretically grounded definition of the affective constructs they used. Despite the methodological impairment, Dunton et al. (2009), McCormick et al. (2008), and Vansteelandt et al. (2007) showed that aPA and positive affect were significantly and positively associated (see Table 2).

**Table 2:** **Either aPA was assessed objectively or affective states were assessed electronically**.

Reference	Aim of the study	Sample	Procedure	Measurements		Results
				Physical activity	Affective states	
Dunton et al. (2009)	To identify cognitive, social, affective, contextual, and physiological antecedents and correlates of physical activity episodes across the day among adults age 50+ years	23 Healthy, community-dwelling older adults (70% female) who did not engage in regular PA, age: *M* = 60.6; SD = 8.2; range = 50–76	Four times a day (fixed interval measurement schedule) across a 2-week period, electronic diaries	Self-report: Pbn were asked whether and how long they had performed each of 12 different types of moderate-to-vigorous activities (MVPA)	Electronic diary; eight different types of emotion (emotionally upset, stressed, lonely, annoyed, tense, sad, frustrated, happy) were assessed with a bipolar scale	Greater levels of positive affect (*t* − 1) predicted higher levels of MVPA and greater levels of negative affect (*t* − 1) predicted lower levels of MVPA
McCormick et al. (2008)	To identify if physical activity level is useful in predicting transitory mood in the everyday lives of people with severe mental illness (SMI)	Individuals with SMI suffer for more than 2 years from severe mental disorders (e.g., bipolar disorder, major depression, schizophrenia). 15 Serbians (age: *M* = 38.9; SD = 11.3) and 22 US citizens (age: *M* = 38.8; SD = 11.4)	Two communities, 7 days, seven times a day (9:00 a.m. to 9:00 p.m.), stratified random schedule	Accelerometer: MTI (7164), right hip, 60 s epoch	Self-report: pager and booklet; positive and negative mood were assessed via dichotomously scored (y/n) mood items: happy, secure, cheerful, bored anxious, angry	Physical activity remained significantly positively associated with mood after accounting for individual variation
Rofey et al. (2010)	Discussion about a pilot study, primarily regarding utilization of ambulatory assessment	20 English Speaking participants, 11–19 years old; BMI *M* = 39, 80% White, 15% African American	14 Cellular phone calls over three extended weekends, they were called twice on weekdays and four times on weekends	Self-report: Pbn reported their physical activity during phone calls Accelerometer; body media, sense wear, weight management system, showing steps taken and calories burned, wearing on upper arm	Structured interview to evaluate affect, via phone call	Technological devices that gather objective data have reasonably high compliance rates, and inform measurement of treatment outcomes in adolescents who are obese
Vansteelandt et al. (2007)	To assess if there is a positive association between patients’ drive for thinness and their level of physical activity across time To assess the association between patients momentary negative/positive emotional states and their level of physical activity over time	32 Female inpatients with an eating disorder, 15–37 years old *M* = 21.6, SD = 6.7; BMI: *M* = 19.4; SD = 4.4; range = 13.5–32.02	1 Week, nine times a day, stratified random Schedule	Self-report: electronic diary, three items referring to type and intensity	Electronic diary; PANAS	Drive for thinness as well as positive emotional states are both, significantly related to patients physical activity. Negative emotional state was not significantly associated with physical activity

### aPa was not assessed objectively, nor were affective states assessed with electronic devices

Most of the selected studies in this section used pagers and booklets to assess aPA and momentary affective states during everyday life, meaning that a pager rang several times a day at a stratified random schedule. The participants had to fill in a booklet as soon as they heard the acoustic signal. Thus, aPA was not assessed objectively, and it was not possible to determine the time point at which the participants actually filled in the questionnaires. Given that the exact time at which each assessment was completed cannot be verified using a paper diary, concerns regarding patient compliance with paper diaries remain.

This section includes 11 studies showing mixed support for the association of aPA and momentary affective states. Eight studies (Gauvin et al., 1996, 2000; Carels et al., 2007; Hausenblas et al., 2008; Bohnert et al., 2009; Kanning and Schlicht, 2010; LePage and Crowther, 2010; Wichers et al., 2011) indicated significant and positive effects of aPA on momentary affective states, whereas three studies (Gauvin and Szabo, 1992; Vendrig and Lousberg, 1997; Focht et al., 2004) did not find significant associations between momentary affective states and aPA. Most studies assessed affective states with validated instruments, such as the PANAS (see Watson and Telegen, 1988; LePage and Crowther, 2010; Wichers et al., 2011), the Exercise-Induced Feeling Inventory (EFI, see Gauvin and Rejeski, 1993; Focht et al., 2004; Hausenblas et al., 2008), the Multidimensional Mood Questionnaire (MDBF, see Steyer et al., 1997; Kanning and Schlicht, 2010), or the Feeling Scale (see Hardy and Rejeski, 1989; Carels et al., 2007).

Nevertheless, several studies analyzed interesting research questions concerning the association between physical activity and momentary affective states in daily life. Gauvin and Szabo (1992) examined the effect of 1-week exercise withdrawal on daily positive and negative affect. Their results showed that exercise withdrawal had no significant impacts on affect. However, the authors did not assess aPA during the study period; thus, they were not able to exclude the possibility that the participants were physically active during the days they were not supposed to be physically active (cf. Hausenblas et al., 2008). Another interesting issue relates to the duration of affect enhancement after being physically active. Wichers et al. (2011) examined changes in affective states before and after daily life increases in aPA. Female twins (*N* = 504, *M*_age_ = 27) were assessed on 5 days with a maximum of 10 measurements per day. The participants filled in a booklet with data regarding aPA (single item) and affect (PANAS) after receiving a beep from a watch. Participants showed higher scores of positive affect after having been physically active. The increase remained significant up to 180 min following the increase in aPA. The authors did not find significant associations with negative affect (see Table 3).

**Table 3:** **aPa was not assessed objectively, nor were affective states assessed electronically**.

Reference	Aim of the study	Sample	Procedures	Measurements		Results
				Physical activity	Affective states	
Bohnert et al. (2009)	To assess if more involvement in active structured discretionary activities would be associated with fewer depressive symptoms and delinquency To assess if positive effect mediate these relationship	246 Urban African American adolescents, 107 boys, 139 girls, 10–15 years *M* = 11.95, SD = 1.23	1 Week/seven times a day	Self-report: pager and booklet; open-ended question “what were you doing?”	Booklet: how were you feeling? – bipolar scale with the following pairs of adjectives: happy – unhappy, weak – strong, angry – friendly, awake – tired, cheerful – grouchy/cranky	Contrary to the expectations, results suggest that involvement in structured discretionary time activities was not associated with less depressive symptoms and delinquency. But more time spent in these activities was positively associated with positive affect
Carels et al. (2007)	To assess the influence of morning mood on exercise, intensity, and duration To assess the effect of exercise intensity and duration on post-exercise mood enhancement	51 Adults (89% female), 31–65 years, *M* = 49.3, SD = 11.2; BMI: *M* = 41.5, SD = 7.3	During the first 4 and the final 4 weeks of a weight loss intervention program participants completed an exercise and mood diary. Participants reported morning, evening, pre- and post-exercise mood, as well as the type, intensity, and duration of exercise	Self-report, booklet: type, duration, intensity	Booklet: feeling scale (single item)	Morning mood was associated with an increased likelihood of exercising; morning mood was not a significant predictor of exercise intensity and duration. Mood ratings were higher following exercise of greater intensity and duration
Focht et al. (2004)	To examine feeling state fluctuations in older, obese adults with knee osteoarthritis throughout the day and in response to an acute bout of physical activity	32 Clinically obese, sedentary adults (25 female) over 60 years of age (*M* = 69.1; SD = 6.5) BMI: *M* = 27.5, with knee osteoarthritis	6 Days, 8:00 a.m. to 9:30 p.m., stratified random schedule; days were divided in three exercise and three non-exercise days	Exercise sessions (11:00 a.m. to 12:00 p.m.) for all participants, walking phase 50–70% of heart rate reserve	Pager and booklet; Exercise-Induced Feeling Inventory (EFI)	Affective states did not change with involvement in acute exercise
Gauvin and Szabo (1992)	To examine the effects of 1-week exercise withdrawal on daily positive and negative affect	12 Experimental and 9 control subjects (14 male), age: *M* = 23.6; SD = 5.4, exercising on average for 7.5 (SD = 3.1) hours/week during the past 5 month	5 Weeks, four times a day (fixed interval measurement schedule); experimental group: participants were asked to stop exercising on day 15 until day 21	Physical activity was not measured during experimental period	Pager and booklet; four positive and five negative affective states: happy, pleased, joyful, enjoyment, unhappy, depressed, blue, angry, frustrated, anxious; and two additional indicators: stressed, relaxed	Exercise withdrawal had no significant impact on positive and negative affect
Gauvin et al. (1996)	To describe changes in affect and feeling states in a community sample of physically active women after acute bouts of vigorous physical activity To explore the moderating role of average mood states, pre-activity scores, estimated aerobic capacity, and frequency of participation in physical activity in the outcome measures	108 Women attending fitness classes at local YMCAs; 86 participants (age: *M* = 33) provided sufficient data	6 Weeks, four times a day, pager signal at random intervals between 8:00 a.m. and 10:00 p.m., additional before and after physical activity bouts lasting 20 min or longer	Self-report: pager and booklet; vigorous (e.g., fitness class or brisk walk) versus light (e.g., leisurely walk or yoga) physical activity	Booklet; affective states were measured with (1) positive affects: happy, pleased, joyful, experience of enjoyment-fun; negative affect: depressed-blue, worried-anxious, frustrated, unhappy, angry-hostile and (2) with Exercise-Induced Feeling Inventory (EFI)	Involvement in physical activity was associated with increases in tranquility, revitalization, and positive affect, and decreases in negative affect. Between-subjects variables did not systematically moderate pre-activity to post-activity changes in mood
Gauvin et al. (2000)	To examine diurnal patterns of feeling states in a community sample of physically active women on days that they were either active of inactive	84 Physically active women from local YMCAs, 19–57 years old *M* = 33.1; SD = 10.4	6 Weeks, four times a day at random intervals between 8:00 a.m. and 10:00 p.m., additional experience sampling questionnaires before and after physical activity bouts lasting 20 min	Self-report: pager and booklet; participants wrote down what they were doing, when responding to the pager call, or before/after exercise	Booklet; Exercise-Induced Feeling Inventory (EFI)	Scores for positive engagement, revitalization, and tranquility assessed following exercise were significantly higher than values recorded at the same time on inactive days
Hausenblas et al. (2008)	To determine if deprivation from exercise resulted in variations in feeling states	40 University students (26 female), age: *M* = 20.5; SD = 2.5, engaged in five weekly bouts of moderate/strenuous exercise	6-Day study, three exercise + three non-exercise days, three times a day (9:00 a.m. to 9:00 p.m.), in addition: immediately before and following exercise	Self-report: pager and booklet; Leisure Time Exercise Questionnaire (LTEQ)	Booklet; Exercise-Induced Feeling Inventory (EFI)	Acute exercise resulted in improved feeling states
Kanning and Schlicht (2010)	To analyze if physically active episodes are associated with more positive mood compared with episodes of inactivity	13 Participants 52–59 years old (9 female)	10-Week period, one to three self-selected episodes per day during their daily routine (defined as occurrences with a definite start/end)	Self-report: booklet; activity described by the participants (strolling around, reading etc.)	Booklet; German-language Multidimensional Mood Questionnaire (MDBF)	When activity increased, valence, energetic arousal, and calmness also increased
LePage and Crowther (2010)	To assess if low trait body dissatisfied individuals will exhibit less state body dissatisfaction and negative affect and more positive affect following exercise than at other times throughout the day	61 Female undergraduate university students, age: *M* = 19.1; SD = 2.88 BMI: *M* = 23.23; SD = 3.65	10 Days, four times throughout the day and following exercise	Self-report; pager and booklet, questions assessing type and amount of exercise	Booklet; ten items of the PANAS-X	Individuals reported less negative affect after exercising than at random assessments
Vendrig and Lousberg (1997)	An initial effort to examine within person relationships among pain intensity, mood, and activity level of chronic pain patients using experience sampling method	57 (31 Females) chronic pain patients, 21–65 years old *M* = 42.3, SD = 10	6 Days/eight times a day, between 8:30 a.m. and 10:30 p.m. (Tuesday–Sunday). one booklet per day	Self-report: pager and booklet; single item scoring activity intensity: 1 = rest, 2 = lying down, 3 = doing nothing to 7 = heavy physical work	Single item: seven point Likert scale, very negative mood to very positive mood	There was no significant relation between activity level and mood
Wichers et al. (2011)	To examine the subtle hour-to-hour fluctuations in affect in relation to increases in physical activity in the flow of daily life	504 Female twins, 18–46 years old; *M* = 27, SD = 7.6	Five consecutive days, ten times a day, between 7:30 and 22:30, stratified random schedule	Self-report: pager and booklet; single item scoring activity intensity from 1 (e.g., resting) to 7 (e.g., running)	Booklet; PANAS	Significant increase in positive affect following the moment of increase in physical activity was replicated across both samples up to 180 min after physical activity. There was no effect of physical activity on negative affect

All in all, half of the studies (11 publications) were of low and the other half of the studies (4 + 7 publications) were of higher methodological quality according to our methodological requirements. Especially the publications of higher methodological quality reported consistently positive association between aPA and momentary affective states in every day life. Only the studies that did not assess aPA objectively and momentary affective states not with electronic devices reported mixed support for the association of affective states and aPA in every day life.

## Studies on Within-Subject Associations between Physical Activity and Affective States in Everyday Life: Discussion, Limitations, and Outlook

As seen from this literature overview, there is strong research interest in within-subject associations assessing the dynamic interactions of momentary affective states and physical activity in everyday life. What is remarkable about the studies presented here is the consistency of their findings within the studies of higher methodological quality. All of these studies (11 out of 22 publications) showed consistently a positive association between aPA and positive affective states. The remaining 11 studies were of lower methodological quality showing mixed support for this association. Because the findings did not show consistent associations between negative affective states and aPA in every day life, the findings did not support the hypothesis that aPA might be a useful strategy to influence person’s affective states in everyday life.

All in all, the methodological quality of most of the studies could be improved. About 30% of the studies used an ambulatory assessment design, assessing physical activity objectively and momentary affective states with electronic devices. Furthermore, 55% of the studies used theoretically grounded definitions of affective constructs.

Three studies explicitly examined the dynamic nature of the aPA-affect relationship, but the results were inconsistent. Schwerdtfeger et al. (2010) and Powell et al. (2009) both reported significant effects on positive affective states, and they indicated that increased levels of affective states predicted increased activity levels, suggesting that momentary affective states and aPA have circular effects on each other. Carels et al. (2007) examined the dynamic process in a behavioral weight loss program for obese people. In this study, thirty-six overweight participants (*M*_age_ = 49, *M*_BMI_ = 41.5) completed an exercise and mood diary during the first 4 weeks and the last 4 weeks of the intervention program. The within-subject effects showed that greater reported exercise intensity and duration were related to greater mood enhancement. Conversely, positive morning mood was associated with an increased likelihood of exercising during the day, but negative morning mood was related to fewer exercises during the day. In contrast, Schwerdtfeger et al. (2010) and Powell et al. (2009) showed that participants with higher scores on negative affective states were more active over the following interval. Thus, participants with high levels of negative affect might have used aPA as a strategy to improve their mood, believing that being more active would diminish their negative affect.

However, the literature overview also revealed inconsistent findings about the impact of aPA on negative affect. Five studies (Vansteelandt et al., 2007; Schwerdtfeger et al., 2008, 2010; Powell et al., 2009; Wichers et al., 2011) did not find a significant effect of aPA on negative affect, whereas two studies (LePage and Crowther, 2010; Dunton et al., 2011) found significant associations.

All in all, the findings did not consistently show that aPA is a “mood repair” strategy that works satisfactorily. The dynamic process of the association between aPA and momentary affective states needs more consideration in further studies assessing whether individuals might use aPA as a means to improve their affective states and regulate their mood.

In addition, more studies are needed that address moderating factors like the dose of physical activity, the quality of affective states, or the timeframe of the association between physical activity and momentary affective states (within-subject analysis). We can show that physical activity may affect energetic arousal immediately but that there seems to be a timeframe for the effect on calmness (Kanning et al., 2012). With more studies of high methodological quality, it may be possible to conduct a meta-analysis of within-subject associations between aPA and affective states in the future.

Furthermore, we found some studies that focused on the effects of physical activity on affect for children and young adults, while others focused on different patient groups. Future studies are needed in these areas, and it would be interesting to discover whether the effect of physical activity on affective states is the same in different age groups or for different types of persons (e.g., patents versus non-patients – a between-subjects analysis).

The findings of our literature overview are inconsistent concerning the dynamic process and the impact of activity on negative affect. Inconsistent findings may be due to the low numbers of studied episodes with high levels of physical activity. McCormick et al. (2008) discuss this problem in their study, which challenges most researchers analyzing the association of affective states and physical activity in everyday life. The authors mentioned an “extreme skewness in the PA variable, with its high proportion of low activity level” (p. 533). Similarly, Dunton et al. (2011, p. S107) determined that they were not able to capture all bouts of physical activity due to the signal-contingent sampling protocol they used. This lack of information is due to the modern condition that the majority of individuals in contemporary societies fail to achieve the recommended minimum level of physical activity (World Health Organization, 2002). Epidemiological research has resulted in a recommendation to be physically active at a moderate intensity for at least 150 min per week (Haskell et al., 2007). Despite these recommendations, the volume of physical activity actually performed by adults and even in children and youth is insufficient, connoting that people are physically active for approximately 3% of their waking hours. It is difficult and improbable to capture these few episodes of physical activity with a time-based or signal-contingent sampling protocol.

To solve this problem, researchers may assess affective states not with a time-based protocol but as a function of predefined intensities of physical activity. Thus, the intensity of the activity triggers the entry of the electronic diary (i.e., interactive ambulatory assessment). Interactive ambulatory assessment is typically employed to maximize variance by physiology- or context-triggered sampling (Intille, 2007). The best-known representative of interactive assessment is Myrtek (2004), who used and validated this method in a series of studies based on many different samples and approximately 1,300 participants. In short, these studies monitored heart rate and physical activity in daily life and separated out in real time heart rate increases caused by physical activity. Any remaining additional heart rate increase was assumed to indicate momentary emotional activation. A recorder/analyzer was programed to trigger a hand-held PC, which in turn signaled the participant to self-report on their current activity, the situation, and their emotions. This phenomenon occurred when the heart rate increase exceeded a certain threshold. With this approach, e-diary prompts were not delivered randomly but during the episodes of interest, therefore maximizing the variance in physiological episodes during e-diary prompts. For an example of how interactive ambulatory assessment can improve the assessment of the relationship between physical activity and momentary affective states, please see Ebner-Priemer et al. (2013).

Despite the methodological benefits, ambulatory assessment has its limitations. Because the strength of an ambulatory assessment approach is its high external validity, experimental control of confounding variables is almost impossible. However, statistical control using self-reports of contextual information and assessment of various confounding effects, such as temperature, physical activity, and breathing patterns, have been successfully employed in ambulatory assessment. We believe that developments in sensor and telecommunication technology will create even more opportunities and applications for research in the near future (Intille, 2007). Data analysis in ambulatory assessment is not trivial, as the data mostly show a hierarchical structure in which multiple assessment points are nested within subjects (Wilhelm, 2001; Bolger et al., 2003; Schwartz and Stone, 2007; Nezlek, 2012). However, there are increasing numbers of published papers on specific aspects of ambulatory assessment analyses (Kubiak and Jonas, 2007; Jahng et al., 2008; Ebner-Priemer and Bussmann, 2011), as well as approaches to calculate the reliability, validity, and sensitivity of change for multilevel data (Wilhelm and Schoebi, 2007). There are also several papers on design issues, which will help novices in this field (Fahrenberg and Myrtek, 2001; Fahrenberg et al., 2007; Piasecki et al., 2007; Shiffman, 2007; Conner and Lehman, 2012), and overviews on hard- and software solutions for ambulatory assessment (Ebner-Priemer and Kubiak, 2007).

## Future Prospects for Intervention Studies

Capturing data in real time could also provide a basis for intervening effectively and efficiently in the often sedentary and inactive lives of most people in modern societies. Such intervention would help to reduce health risks and to promote mental health. For example, Wen et al. (2011) conducted a prospective cohort study with a sample of more than 400,000 individuals and a follow-up period of 8 years. The authors showed that individuals who were physically active for 15 min per day had a 14% reduction in the risk of all-cause mortality. A person can be physically active for 15 min if he or she exercises or regularly participates in a sport. However, it is also possible to perform daily physical activities that are spontaneous or habitually performed, such as taking a stroll, transporting oneself to work, or doing chores. These aPA should be classified as significant preventative health behaviors.

Ambulatory Assessment Interventions (AAIs) with individually tailored, moment-specific feedback have the potential to influence and support the individual when the unhealthy behavior (e.g., inactivity, sedentariness, unhealthy food intake) actually occurs. Individually tailored, moment-specific feedback added to an ambulatory assessment clearly leaves pure assessment behind and is used as a treatment component. Such online feedback can urge participants to perform physical activity or to disrupt episodes of sedentariness. From a learning perspective, this method should be superior to traditional interventions in which the advantages of regular physical activity are explained in a single session. In a standard intervention, the challenge is to generalize behavior that was learned in a certain setting to everyday life. In an AAI with individually tailored, moment-specific feedback, components are delivered in daily life directly in the situations in which participants should change their behavior. For instance, a person enters information about how, when, and where he would like to be physically active into a smartphone. The smartphone reminds the person of his or her activity goals and may provide alternative activity programs or information about the opening hours of a fitness center nearby. For a more detailed description of such an intervention, please see Schwerdtfeger et al. (2012).

Heron and Smyth (2010) listed in their review 27 studies using an AAI targeting a variety of psychological and physical symptoms and health/disease behaviors (e.g., smoking cessation, weight loss, diabetes management, alcohol use, healthy eating, physical activity). The review provided a more stringent test of the efficacy of AAI compared to previously validated interventions (e.g., psychoeducation). The review also showed that an AAI intervention is not effective only for younger people. Younger people are mostly familiar with hand-held computers and smartphones; thus, an intervention using this technology may fit better within their lifestyle, potentially making AAI more readily accepted and improving the chances of lasting behavioral changes. However, the review listed three intervention studies with people older than 55 years showing that the intervention is effectively implemented and accepted by these older people.

Implementing interventions in everyday life presents particular challenges with respect to intervention development, planning, and compliance, but AAI offers the chance to support the individual exactly at the moment when he or she requires help.

## Conclusion

Analyzing within-subject associations with less methodological impairment will provide a deeper understanding of the dynamic linkage between momentary affective states and physical activity in everyday life. We recommended three methodological requirements for future studies: first, assessments should be done repeatedly in real time. Second, physical activity should be measured objectively. Third, momentary affective states should be measured electronically.

To analyze the contribution of physical activity in everyday life to the variability of affective states would broaden our knowledge of the mental health benefits of such activity. This knowledge would help establish appropriate physical activity guidelines and support ambulatory assessment treatment as a proactive intervention in a person’s life.

## Conflict of Interest Statement

The authors declare that the research was conducted in the absence of any commercial or financial relationships that could be construed as a potential conflict of interest.
